# Phosphorylated 4EBP1 is associated with tumor progression and poor prognosis in Xp11.2 translocation renal cell carcinoma

**DOI:** 10.1038/srep23594

**Published:** 2016-03-30

**Authors:** Yuanyuan Qu, Rui Zhao, Hongkai Wang, Kun Chang, Xiaoqun Yang, Xiaoyan Zhou, Bo Dai, Yao Zhu, Guohai Shi, Hailiang Zhang, Dingwei Ye

**Affiliations:** 1 Department of Urology, Fudan University Shanghai Cancer Center, Shanghai, 200032, China; 2 Institute of Biomedical Sciences, Fudan University, Shanghai, 200032, China; 3 Department of Pathology, Fudan University Shanghai Cancer Center, Shanghai, 200032, China; 4 Department of Oncology, Shanghai Medical College, Fudan University, Shanghai, 200032, China

## Abstract

Two main signaling pathways, PI3K-AKT-mTOR and RAS-MAPK, are involved in transmitting the proliferative signals which play critical roles in human cancers. However, the functions of these pathways in Xp11.2 RCC remain undefined. This study aimed to explore the expression of mTOR and MAPK cascades in Xp11.2 RCC and to assess the prognostic significance of proteins evaluated. Immunohistochemistry was performed to evaluate the expression of 4EBP1, p-4EBP1, p-mTOR, p-S6K and p-MAPK in 36 adult Xp11.2 RCC patients who were confirmed by FISH assay. Cox regression models were used to evaluate the prognostic value of all covariates. Among 36 assessed patients, 14 (38.9%), 26 (72.2%), 16 (44.4%), 19 (52.8%), and 9 (25.0%) patients showed high expression of 4EBP1, p-4EBP1, p-mTOR, p-S6K, and p-MAPK, respectively. We noted that p-4EBP1 expression was significantly correlated with lymph node metastases (*P* = 0.027). Multivariate analysis showed that high p-4EBP1 expression was an independent adverse prognostic factor for both PFS (HR = 33.750, *P* = 0.017) and OS (HR = 56.111, *P* = 0.026). Our findings suggest that p-4EBP1 may serve as a funnel factor that converge the upstream proliferative oncogenic signals. Effective inhibition of the pathways responsible for 4E-BP1 phosphorylation might be a useful strategy to improve the outcome of Xp11.2 RCC patients.

Renal cell carcinoma (RCC) is the most common type of kidney cancer in adults and accounts for approximately 90% of all adult renal malignancies and 2–3% of all cancers^1^,^2^. RCC is a heterogeneous tumor and can be histologically classified into several subtypes, among which clear cell (70–80%), papillary (10–15%), and chromophobe (4–5%) are the most prevalent types, and each has specific histopathological and genetic characteristics. Xp11.2 translocation renal cell carcinoma (Xp11.2 RCC), a rare subtype of RCC, was first delineated as a genetically distinct disease entity in the 2004 World Health Organization (WHO) renal tumor classification scheme^3^. Xp11.2 RCC is featured by different translocations involving chromosome Xp11.2, all of which result in transcription factor E3 (TFE3) gene fusions. So far, at least 5 fusions of TFE3 gene with different partners have been identified including PRCC (1q21), ASPL (17q25), PSF (1q34), CLTC (17q23), and NonO (Xq12)^4^,^5^,^6^,^7^,^8^.

Despite Xp11.2 RCC predominantly occurs in children and adolescents, adult Xp11.2 RCC patients may vastly outnumber pediatric patients due to much higher incidence of RCC in adult population^9^. Xp11.2 RCC, especially in adult patients, often present at advanced stage and demonstrate an invasive clinical course^10^,^11^. Although the development of multimodality therapies including surgery, immunotherapy, and vascular endothelial growth factor (VEGF)-targeted therapy, prognosis of adult Xp11.2 RCC patients remains poor^10^,^11^. Identification of reliable prognostic molecular markers for Xp11.2 RCC could provide novel therapeutic targets, which is critically important for developing molecular-profile-directed therapy. Currently, there is no molecular marker available to predict the outcome of Xp11.2 RCC.

The activation of cell signaling pathways involved in cell growth and proliferation plays a critical role in human cancers^12^. Two main signaling pathways, phosphatidylinositol–3 kinase (PI3K)-protein kinase B (AKT)-mammalian target of rapamycin (mTOR) and RAS-mitogen-activated protein kinase (MAPK) pathways, are involved in transmitting the proliferative signal from the membrane receptors to the nucleus and driving cell proliferation^13^,^14^. A final effector of these signaling cascades is the cap-dependent mRNA translation initiation complex, which is negatively regulated by phosphorylated eukaryotic initiation factor 4E (eIF4E) binding protein 1 (p-4EBP1)^13^,^14^,^15^. Furthermore, p-4EBP1 has recently been reported to correlate with aggressive pathologic grade and inferior prognosis regardless of the upstream oncogenic alterations in a great deal of malignancies including RCC, endometrial carcinoma, breast cancer, ovarian cancer, melanoma, cervical carcinoma, astrocytoma, esophageal squamous cell carcinoma and hilar cholangiocarcinoma^13^,^14^,^16^,^17^,^18^,^19^,^20^,^21^,^22^,^23^. Under the circumstances, we hypothesize that p-4EBP1 may act as a “bottleneck” or funnel factor that converge upstream oncogenic signals and drive the proliferative signal downstream.

To investigate this hypothesis, activation of the two signaling pathways and downstream factors, including phosphorylated mTOR (p-mTOR), phosphorylated MAPK (p-MAPK), 4EBP1, p-4EBP1 and phosphorylated ribosomal protein S6 kinase (p-S6K), were detected using immunohistochemistry in 36 adult Xp11.2 RCC patients who were confirmed by the TFE3 break-apart fluorescence *in situ* hybridization (FISH) assay. Moreover, we evaluated the correlation between expression of these proteins and patient clinicopathological characteristics and assessed the prognostic value of these factors for progression-free survival (PFS) and overall survival (OS). To the best of our knowledge, this is the first study from ethnic Chinese population to explore the prognostic factors for Xp11.2 RCC patients.

## Results

### Patient characteristics

**Figure 1:**
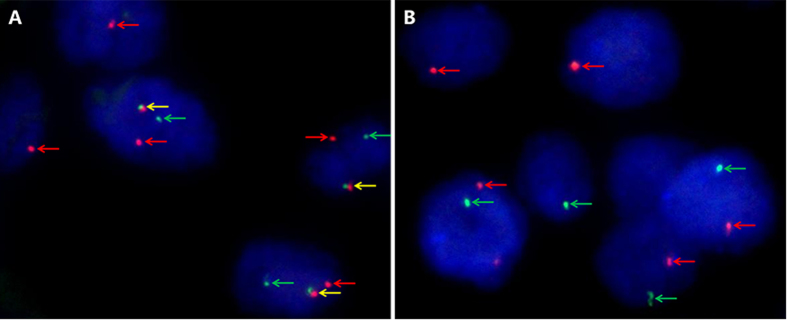
Representative images of the TFE3 break-apart probe assay. (**A**) Demonstrate a pair of split red and green signals (red and green arrows) as well as a normal fused hybridization signals (yellow arrows) in a female patient, indicating the translocation of one X chromosome and a normal another (×1000); (**B**) Demonstrate a pair of split red and green signals (red and green arrows) in a male patient, indicating the translocation of the only X chromosome (×1000). TFE3, transcription factor E3.

Representative images of the TFE3 break-apart FISH assay are demonstrated in Fig. 1, which indicate the existence of TFE3 rearrangement associated with Xp11.2 translocation. Follow-up continued until 30 October 2015, with the median duration of 30 months (range, 2–87 months). Of the 36 enrolled patients, 11 (30.6%) died of the disease and 11 (30.6%) survived with progressive disease at the time of last follow-up. According to RECIST criteria, a partial response was observed in 3 of 18 (16.7%) metastatic Xp11.2 RCC patients who underwent VEGF-targeted therapy. Among the 4 patients who received second-line everolimus therapy, 2 patients achieved partial remission with the duration of 4 months and 8 months, respectively, one patient achieved stable disease for 5 months, and the other patient was primarily resistant to everolimus therapy. The median PFS and OS for the entire cohort were 13.0 months (95% confidence interval [CI], 9.4–16.6) and 36.0 months (95% CI, 23.9–48.1), respectively. The clinicopathological characteristics of the entire cohort are summarized in Table 1.

### Protein expression profile

**Figure 2:**
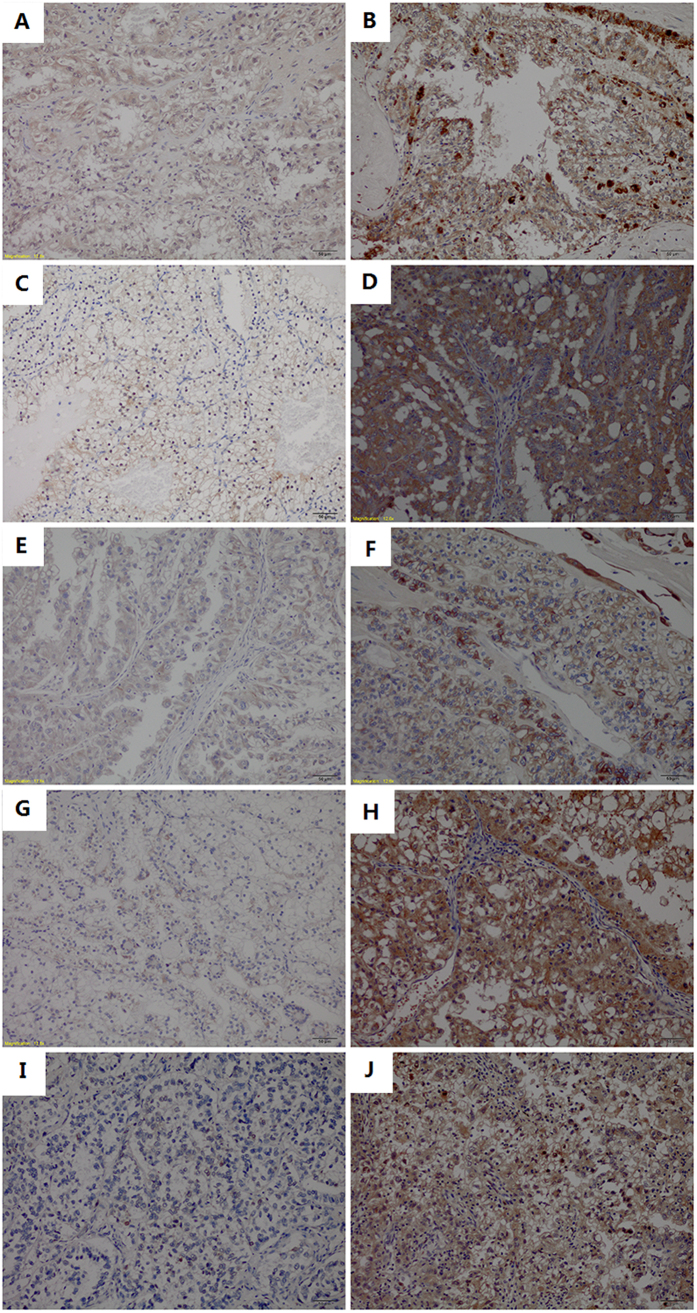
Representative images of immunohistochemical staining. (**A**) Low expression of 4EBP1; (**B**) High expression of 4EBP1; (**C**) Low expression of p-4EBP1; (**D**) High expression of p-4EBP1; (**E**) Low expression of p-mTOR; (**F**) High expression of p-mTOR; (**G**) Low expression of p-S6K; (**H**) High expression of p-S6K (**I**) Low expression of p-MAPK; (**J**) High expression of p-MAPK. 4EBP1, eukaryotic initiation factor 4E (eIF4E) binding protein 1; p-4EBP1, phosphorylated eukaryotic initiation factor 4E (eIF4E) binding protein 1; p-mTOR, phosphorylated mammalian target of rapamycin; p-S6K, phosphorylated ribosomal protein S6 kinase; p-MAPK, phosphorylated mitogen-activated protein kinase.

Immunohistochemical staining showed that 4EBP1, p-4EBP1 and p-MAPK expressed either in the cytoplasm or in the nucleus, while the predominant pattern of p-mTOR and p-S6K staining was cytoplasmic. The expression profile of proteins under evaluation are displayed in Table 2. Among 36 assessed Xp11.2 RCC patients, 14 (38.9%), 26 (72.2%), 16 (44.4%), 19 (52.8%), and 9 (25.0%) patients showed high expression of 4EBP1, p-4EBP1, p-mTOR, p-S6K, and p-MAPK, respectively. Encouragingly, we found 21 of 26 (80.8%) patients with high expression of p-4EBP1 exhibited either high p-mTOR expression or high p-MAPK expression, which is in accordance with the fact that both PI3K-AKT-mTOR and RAS-MAPK pathways mediate phosphorylation of 4EBP1. Representative images of immunohistochemical staining for these proteins are showed in Fig. 2. More interestingly, among the four patients underwent second-line everolimus therapy, the two patients who achieved partial remission both showed high expression of p-4EBP1 and p-mTOR, the patient who achieved stable disease showed high expression of p-4EBP1 and the patient who had progressed disease showed high expression of 4EBP1.

### Correlations between expression profile and clinicopathological characteristics

The clinicopathological characteristics of patients grouped by p-4EBP1 expression level are listed in Table 1. A significant correlation between p-4EBP1 expression and presence of positive lymph nodes was observed (*P* = 0.027). The incidence of positive lymph nodes was 50.0% in patients with high expression of p-4EBP1, which was higher than that in patients with low expression of p-4EBP1 (10.0%). We found no significant association of 4EBP1, p-mTOR, p-S6K and p-MAPK expression with any clinicopathological features including age at surgery, sex, clinical manifestation, tumor location, surgery procedure, tumor size, tumor stage and International Society of Urological Pathology (ISUP) grade (Supplementary Table 1).

### High p-4EBP1 expression was associated with tumor progression and poor prognosis

**Figure 3:**
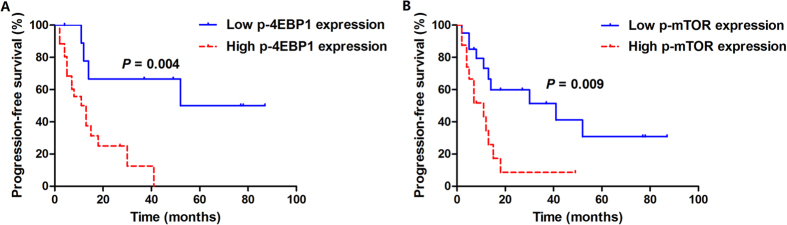
Kaplan–Meier analysis of PFS curves in 36 adult Xp11.2 RCC patients. (**A**) Stratified according to p-4EBP1 expression (log-rank *P* = 0.004); (**B**) Stratified according to p-mTOR expression (log-rank *P* = 0.009). PFS, progression-free survival; Xp11.2 RCC, Xp11.2 translocation renal cell carcinoma; p-4EBP1, phosphorylated eukaryotic initiation factor 4E (eIF4E) binding protein 1; p-mTOR, phosphorylated mammalian target of rapamycin.

**Figure 4:**
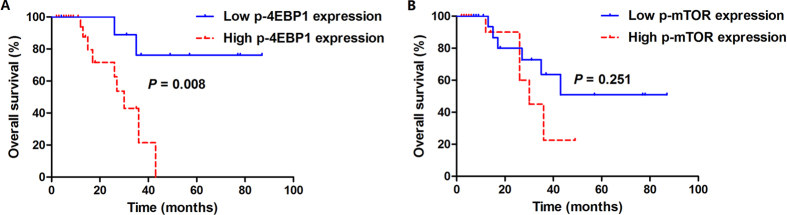
Kaplan–Meier analysis of OS curves in 36 adult Xp11.2 RCC patients. (**A**) Stratified according to p-4EBP1 expression (log-rank *P* = 0.008). (**B**) Stratified according to p-mTOR expression (log-rank *P* = 0.251). OS, overall survival; Xp11.2 RCC, Xp11.2 translocation renal cell carcinoma; p-4EBP1, phosphorylated eukaryotic initiation factor 4E (eIF4E) binding protein 1; p-mTOR, phosphorylated mammalian target of rapamycin.

In survival analysis in relation to the immunohistochemical expression of proteins examined, only p-4EBP1 and p-mTOR were significantly associated with worse progression-free survival (PFS). With regard to p-4EBP1, 69.2% (18/26) of high expression cases experienced disease progression with a median PFS of 12 months (95% CI, 7.1–18.9), while only 40% (4/10) of low expression cases experienced disease progression with 52 months of median PFS (log-rank *P* = 0.004). Regarding p-mTOR, there were 12 cases with progressive disease (75%) in high expression cohort with a median PFS of 11 months (95% CI, 4.2–17.8), and only 10 cases with progressive disease (50%) in low expression cohort and the median PFS was 41 months (95% CI, 2.0–80.0, log-rank *P* = 0.009). The Kaplan-Meier survival curves for PFS according to expression of proteins studied are displayed in Fig. 3 and supplementary Fig. 1. When overall survival (OS) was assessed, high p-4EBP1 expression significantly correlated with inferior survival (*P* = 0.008). 34.6% (9/26) of high p-4EBP1 expression cases died of the disease with a median OS of 30 months (95% CI, 24.1–35.9), whereas only 20% (2/10) low p-4EBP1 expression cases died of the disease and the median OS had not been reached. No significant association between other protein markers evaluated and OS was identified. The time distributions according to expression of proteins studied with respect to OS are given in Fig. 4 and supplementary Fig. 2.

Using the Cox proportional hazards model, univariate and multivariate analyses were performed to assess the independent prognostic value of clinicopathological and immunohistochemical parameters for PFS and OS. The results of Cox regression analyses for PFS and OS are depicted in Tables 3 and 4, respectively. In multivariate analyses with regard to PFS, p-4EBP1 (HR = 33.750, 95% CI, 1.883–604.899, *P* = 0.017) and p-mTOR (HR = 8.766, 95% CI, 1.212–63.424, *P* = 0.032), along with the established indicators M stage (HR = 28.727, 95% CI, 3.079–267.990, *P* = 0.003) and N stage (HR = 1.340, 95% CI, 1.007–1.784, *P* = 0.045), showed independent prognostic significance after adjusting for covariates (Table 3). Remarkably, p-4EBP1 (HR = 56.111, 95% CI, 1.600–1967.704, *P* = 0.026) and M stage (HR = 19.849, 95% CI, 1.121–351.393, *P* = 0.042) remained as independent adverse prognostic factors for OS in multivariate analyses (Table 4).

## Discussion

The present study is the first to examine the cell signaling pathways, including mTOR and MAPK cascades, in a series of adult Xp11.2 RCC patients, and to correlate expression patterns with clinicopathological characteristics and patient outcome to assess the prognostic significance of proteins evaluated. In this study, we noted that high p-mTOR expression was correlated with tumor progression. More importantly, we observed that high expression of p-4EBP1 in primary tumors was significantly associated with lymph node metastases, tumor progression and an adverse prognosis regardless of the upstream oncogenic alterations. Our findings suggest that p-4EBP1 seems act as a hallmark or funnel factor that channel the proliferative oncogenic signal and could be a highly relevant molecular marker of malignant potential and a therapeutic target for patients with Xp11.2 RCC.

Growth factor receptors and cell signaling pathways play essential roles in carcinogenesis of human cancers. Activation of membrane growth factor receptors drives cell proliferation signals through at least two major biochemical pathways, PI3K-AKT-mTOR and RAS-MAPK pathways^13^,^14^. The PI3K-AKT pathway canonically regulates translation via activation of mTOR kinase and subsequent phosphorylation of its substrates, 4EBP1 and S6K. The MAPK pathway, which is involved in cell proliferation, survival, apoptosis and metabolism^24^, also contributes to the phosphorylation of 4EBP1^25^. Hence, both PI3K-AKT-mTOR and RAS-MAPK pathways mediate phosphorylation of 4EBP1, which leads to formation of the cap-dependent mRNA translation initiation complex^15^,^25^,^26^. During cap-dependent translation, eIF4E binds to the mRNA cap structure and promotes formation of the eIF4E initiation complex and ribosome binding. When 4EBP1, an eIF4E-binding protein, is active (non-p-4EBP1), it binds to eIF4E and hinder formation of the initiation complex and then translation is blocked, favoring apoptosis. Nevertheless, when 4EBP1 is phosphorylated, the affinity for eIF4E binding is reduced, eIF4E is released, and cap-dependent translation can initiate^27^.

The eIF4E plays a central role in the regulation of translation and has an important function in the translation of key proteins in tumor transformation, including cyclin D1, Myc, fibroblastic growth factor, and vascular endothelial growth factor^28^. Avdulov *et al.* reported that eIF4E is an essential component of the malignant phenotype in breast carcinoma and hyperphosphorylation of 4EBP1 is crucial in this effect^29^. In their study, transfer of 4E-BP1 phosphorylation site mutants into breast carcinoma cell suppressed their tumorigenicity, whereas loss of these 4E-BP1 phosphorylation site mutants accompanied spontaneous reversion to a malignant phenotype. Coincidently, results from a recent study on RCC revealed that overexpression of p-4EBP1 and eIF4E synergistically promote disease progression^23^. Moreover, Salehi *et al.* reported that higher eIF4E expression and lower 4EBP1 expression were correlated with advanced stage in esophageal cancer^30^. These findings could be explained by the fact that the phosphorylation of 4EBP1 releases eIF4E and enhances cap-dependent translation in favor of tumor cell growth and proliferation. Our results regarding p-4EBP1 are supported by the oncogenic role of eIF4E. Future studies are warranted to validate eIF4E expression in Xp11.2 RCC as a prognostic factor and to correlate it with 4EBP1.

Aberrant activation of mTOR in relation to poor prognosis has been reported in various cancers^31^,^32^,^33^,^34^. mTOR has been considered as an attractive therapeutic target for cancer therapy in recent years and yielded profound effectiveness. Excitingly, Parikh *et al.* reported that the administration of temsirolimus, an inhibitor of mTOR kinase, to an adult Xp11.2 RCC with extensive metastasis achieved exceptional response^35^. In our study, we noted that high expression of p-mTOR in Xp11.2 RCC was significantly correlated with shorter PFS. More interestingly, the administration of everolimus, an inhibitor of mTOR kinase, in 4 patients who progressed after VEGF-targeted therapy obtained favorable outcomes with 2 patients achieving partial remission with the duration of 4 months and 8 months, respectively, and one patient achieving stable disease for 5 months. These results implied that the activation of mTOR signaling pathway, which is involved in cell growth and proliferation through phosphorylation of 4EBP1 followed by up-regulating translational initiation^36^, might play an essential role in the progression of Xp11.2 RCC. Thus, combined suppression of the upstream signaling pathways that activate 4EBP1 may prove to be an effective strategy to improve treatment outcome of Xp11.2 RCC patients.

Interestingly, we found no correlation of p-S6K expression, which was also activated by mTOR, with patient clinicopathological characteristics and survival and our findings were consistent with previous studies on a variety of malignancies^14^,^16^,^19^. The differences between p-4EBP1 and p-S6K expression found in Xp11.2 RCC patients may be an indication that p-4EBP1 could be phosphorylated by other pathways^15^,^27^ or that mTOR phosphorylates 4EBP1 more actively in Xp11.2 RCC. Results from published studies showed that mTOR-dependent phosphorylation of p-S6K is not linearly correlated with 4EBP1 phosphorylation^37^,^38^, which could partly explain the lack of prognostic value of p-S6K observed in this study.

We acknowledge that there are certain limitations in this study. On the one hand, the sample size was relatively small due to the low incidence of this rare disease. On the other hand, the dual color, break-apart FISH assay employed in our study cannot detect each partner of the specific translocation, although this method serve as a convenient diagnostic tool in formalin-fixed paraffin-embedded tissues and provides a confirmative diagnosis for Xp11.2 RCC in clinical setting. Notwithstanding these limitations, results from the current study underscore that p-4EBP1 may serve as a hallmark or funnel factor where upstream oncogenic signals converge and dephosphorylation of 4E-BP1 could be a powerful therapeutic options in Xp11.2 RCC. Further research of the signaling pathways that regulate 4E-BP1 phosphorylation in Xp11.2 RCC may reveal additional therapeutic strategies to attenuate unrestricted protein biosynthesis in this cancer.

In conclusion, p-4EBP1 is prevalent in Xp11.2 RCC and high p-4EBP1 expression is independently associated with tumor progression and an adverse prognosis. Our findings suggest that p-4EBP1 may serve as a hallmark or funnel factor that converge the upstream proliferative oncogenic signals. Effective inhibition of the pathways responsible for 4E-BP1 phosphorylation might be a useful strategy to improve the outcomes of Xp11.2 RCC patients.

## Materials and Methods

### Patients and tissue samples

The study cohort consisted of 36 adult Xp11.2 RCC patients who underwent radical or partial nephrectomy for the treatment of RCC from January 2008 to June 2015 at Fudan University Shanghai Cancer Center. All the cancers tested were confirmed containing TFE3 rearrangement associated with Xp11.2 translocation by the TFE3 break-apart FISH assay. 6 patients with distant metastases at presentation and another 12 patients who occurred disease progression after surgery received VEGF-targeted therapy (Sorafenib/Sunitinib). Furthermore, everolimus (RAD001), an oral mTOR inhibitor, was administrated to 4 patients who progressed after VEGF-targeted therapy. Clinicopathological characteristics including demographic data, clinical manifestations, surgical techniques, pathological findings, adjuvant therapy, clinical outcomes and follow-up information were collected from a medical record review. Tumor sizes were evaluated by measuring the largest diameter of the tumor mass removed surgically. Tumor grade was assessed in accordance with the 2012 ISUP grading system for RCC. All cases were staged according to the 2010 American Joint Committee on Cancer TNM staging system.

All study samples were obtained FFPE tissue blocks. The hematoxylin and eosin (H&E)-stained sections were independently reviewed by two experienced genitourinary pathologists to determine the presence of representative areas of the original samples and to evaluate the ISUP grade. The present study was carried out in accordance with the ethical standards of Helsinki Declaration II and approved by the Institution Review Board of Fudan University Shanghai Cancer Center. Written informed consent was obtained from each patient before any study-specific investigation was performed.

### TFE3 FISH analysis

A dual-color, break-apart FISH assay was performed to detect TFE3 rearrangement using the TFE3 (Xp11) break probe set (KBI-10741, Poseidon, KREATECH Diagnostics, Amsterdam, Netherlands). The 4-μm-thick, paraffin-embedded sections were deparaffinized, dehydrated, washed twice in distilled water for 2 minutes, and incubated in pretreatment solution (1 M NaSCN) at 80 °C for 30 minutes. Slides were then digested in 0.4 mL pepsin solution at 37 °C for 15 minutes, rinsed twice in 2× sodium saline citrate (SSC) for 5 minutes, fixed in 4% formaldehyde for 10 minutes at room temperature, dehydrated by immersing in 70%, 85%, and 100% ethanol for 1 minute each at room temperature and followed by air-dried. Slides containing probe mixture (10 μL/slide) were incubated in humidified chamber at 75 °C for 5 minutes to denature the probe and target DNA simultaneously and was subsequently incubated at 37 °C overnight for hybridization. The cover slips were removed, and the slides were washed in 0.4 × SSC for 2 minutes at 72 °C followed by a wash with 2 × SSC for 2 minute at room temperature. The nuclei were counterstained with 4,6-diamidino-2-phenylindole (DAPI) and all slides were maintained at 4 °C in the dark.

FISH signals were assessed under an Olympus BX51TRF microscope (Olympus, Japan) equipped with a triple-pass filter (DAPI/Green/Orange; Vysis). Signals were considered to be split when the distance between red and green signals ≥2 signal diameters. Cells without the rearrangement had one (for males) or two (for females) sets of red and green fusion signals indicating intact Xp11. For each case, a minimum of 100 tumor nuclei were evaluated. To avoid false positives due to nuclear truncation, overlapping cells indistinguishable as separate nuclei were not included in the analysis. A positive result was reported when ≥10% of the tumor nuclei had break-apart signals.

### Immunohistochemistry

Immunohistochemistry staining was performed using the avidin-biotin-peroxidase technique. The 4-μm thick sections from representative FFPE blocks were deparaffinized in a series of xylene and rehydrated in a graded series of ethanol solutions. Endogenous peroxidase was quenched in 3% hydrogen peroxide in absolute methanol for 15 minutes at 37 °C Heat-induced antigen retrieval was carried out in 10 mM citrate buffer solution (PH 6.0) and then incubated with the primary antibody at 4 °C overnight. The following primary antibodies were assayed: rabbit anti-total 4E-BP1 monoclonal antibody (#9644, dilution 1:100), rabbit anti-phospho-4E-BP1 (Thr37/46) monoclonal antibody (#2855, dilution 1:300); rabbit anti-phospho-mTOR (Ser2448) monoclonal antibody (#2976, dilution 1:100), rabbit anti phospho-p44/42 MAPK (Erk 1/2) (Thr 202/Tyr204) monoclonal antibody (#4370; dilution 1:100) (Cell Signaling Technology, Danvers, MA, USA) and Rabbit anti-phospho-S6K (T389/412) polyclonal antibody (ab60948; dilution 1:200) (abcam, UK). Immunostaining was performed with the EnVision system (Dako, Cytomation, Glostrup, Denmark). All slides were counterstained with hematoxylin, dehydrated, and mounted. Omission of the primary antibody with phosphate-buffered saline served as a negative control.

All slides were examined and scored by two experienced pathologists, who were blinded to all clinical data, in an open discussion. For the immunoreactivity, a histoscore (H-score) based on the percentage of immunoreactive cells (0–100%) and the intensity of immunostaining (negative; weak: +; moderate: ++; strong: +++) was calculated to group the expression into four categories. The four categories of H-score were defined as negative, weak, moderate, and strong. Tumors with no staining were scored as negative. Tumors with weak intensity (+) in <33% of cells were scored as weak. Tumors with moderate intensity (++) in >66% of cells or strong intensity (+++) in >33% of cells were scored as strong. The remaining tumors were scored as moderate. A tumor with the H-score in the strong category was considered high expression and that with the H-score other than strong category was considered low expression^18^.

### Statistical analysis

Continuous data are presented as median (range) and categorical data are presented as number (proportion). Patient clinicopathological characteristics according to expression of 4EBP1, p-4EBP1, p-mTOR, p-S6K and p-MAPK, respectively, were compared using the Student’s t-test for continuous variables and the Chi-square test for categorical variables. PFS was defined as the time interval between the date of surgery and the date of disease progression or censoring at the time of last follow-up. OS was defined from the initiation of surgery to the date of death or last follow-up, whichever occurred first. PFS and OS were assessed using the Kaplan-Meier method and compared by the log-rank test. Univariate and multivariant Cox regression models were used to evaluate the predictive role of all covariates for PFS and OS. In multivariant Cox regression analyses, covariates included age at surgery, sex, clinical manifestations, tumor location, surgery procedure, tumor size, tumor stage, ISUP grade and expression of proteins under evaluation. All statistical analyses were performed using SPSS software version 16.0 (SPSS Inc., Chicago, IL, USA). The *P* value was two tailed and was considered to be statistically significant when *P* < 0.05.

## Additional Information

**How to cite this article**: Qu, Y. *et al.* Phosphorylated 4EBP1 is associated with tumor progression and poor prognosis in Xp11.2 translocation renal cell carcinoma. *Sci. Rep.* **6**, 23594; doi: 10.1038/srep23594 (2016).

## Supplementary Material

Supplementary Information

## Figures and Tables

**Table 1:** Clinicopathological characteristics in relation to p-4EBP1 expression status.

Variable	Entire group (n = 36)	p-4EBP1 expression		*P* value
		Low expression (n = 10)	High expression (n = 26)	
Median age at surgery (y, range)	29.0 (14.0–63.0)	27.0 (15.0–63.0)	30.0 (14.0–48.0)	0.559
Sex (n, %)				0.763
Male	13 (36.1)	4 (40.0)	9 (34.6)	
Female	23 (63.9)	6 (60.0)	17 (65.4)	
Clinical manifestation (n, %)				0.900
Incidental	15 (41.7)	4 (40.0)	11 (42.3)	
Symptomatic	21 (58.3)	6 (60.0)	15 (57.7)	
Laterality (n, %)				0.137
Left	18 (50.0)	7 (70.0)	11 (42.3)	
Right	18 (50.0)	3 (30.0)	15 (57.7)	
Operation (n, %)				0.739
Radical nephrectomy	30 (83.3)	8 (80.0)	22 (84.6)	
Partial nephrectomy	6 (16.7)	2 (20.0)	4 (15.4)	
Median tumor size (cm, range)	5.7 (2.0–18.0)	7.1 (3.0–18.0)	5.5 (2.0–15.0)	0.190
T stage at presentation (n, %)				0.293
T1–T2	24 (66.7)	8 (80.0)	16 (61.5)	
T3–T4	12 (33.3)	2 (20.0)	10 (38.5)	
N stage at presentation (n, %)				0.027
N0	22 (61.1)	9 (90.0)	13 (50.0)	
N1	14 (38.9)	1 (10.0)	13 (50.0)	
M stage at presentation (n, %)				0.274
M0	28 (77.8)	9 (90.0)	19 (73.1)	
M1	8 (22.2)	1 (10.0)	7 (26.9)	
ISUP grade (n, %)				0.842
1–2	8 (22.2)	2 (20.0)	6 (23.1)	
3–4	28 (77.8)	8 (80.0)	20 (76.9)	
Adjuvant therapy (n, %)				0.080
None	13 (36.1)	6 (60.0)	7 (26.9)	
Immunotherapy	5 (13.9)	2 (20.0)	3 (11.5)	
Targeted therapy	18 (50.0)	2 (20.0)	16 (61.5)	

p-4EBP1, phosphorylated eukaryotic initiation factor 4E (eIF4E) binding protein 1; ISUP, International Society of Urological Pathology.

**Table 2:** Distribution of immunohistochemical staining patterns of the proteins under evaluation across 36 assessed Xp11.2 RCC patients.

Case	4EBP1 expression	p-4EBP1 expression	p-mTOR expression	p-S6K expression	p-MAPK expression
No. of high expression (%)	14 (38.9%)	26 (72.7%)	16 (44.4%)	19 (52.8%)	9 (25.0%)
1	Low	Low	High	High	Low
2	High	Low	Low	Low	Low
3	High	Low	Low	Low	Low
4	High	Low	Low	Low	Low
5	High	Low	Low	Low	Low
6	High	Low	Low	Low	High
7	High	Low	Low	High	Low
8	High	Low	Low	High	Low
9	High	Low	Low	High	Low
10	High	Low	High	High	Low
11	Low	High	Low	Low	Low
12	Low	High	Low	Low	Low
13	Low	High	High	Low	Low
14	Low	High	High	Low	Low
15	Low	High	High	Low	Low
16	Low	High	Low	Low	High
17	Low	High	Low	Low	High
18	Low	High	Low	Low	High
19	Low	High	Low	Low	High
20	Low	High	Low	High	Low
21	Low	High	Low	High	Low
22	Low	High	Low	High	Low
23	Low	High	High	High	Low
24	Low	High	High	High	Low
25	Low	High	High	High	Low
26	Low	High	High	High	Low
27	Low	High	High	High	Low
28	Low	High	High	High	Low
29	Low	High	High	High	Low
30	Low	High	High	High	Low
31	Low	High	Low	High	High
32	High	High	High	Low	Low
33	High	High	Low	Low	High
34	High	High	Low	Low	High
35	High	High	High	High	Low
36	High	High	High	High	High

Xp11.2 RCC, Xp11.2 translocation renal cell carcinoma; 4EBP1, eukaryotic initiation factor 4E (eIF4E) binding protein 1; p-4EBP1, phosphorylated eukaryotic initiation factor 4E (eIF4E) binding protein 1; p-mTOR, phosphorylated mammalian target of rapamycin; p-S6K, phosphorylated ribosomal protein S6 kinase; p-MAPK, phosphorylated mitogen-activated protein kinase.

**Table 3:** Univariate and multivariate Cox regression analyses of PFS in 36 enrolled adult Xp11.2 RCC patients.

Covariates	Univariate analysis		Multivariate analysis	
	HR (95%CI)	P value	HR (95%CI)	P value
Age at surgery	0.959 (0.916–1.003)	0.068	0.960 (0.913–1.010)	0.112
Sex (male vs. female)	0.633 (0.268–1.494)	0.297	0.964 (0.159–5.834)	0.968
Clinical manifestation (incidental vs. symptomatic)	0.940 (0.395–2.235)	0.888	0.960 (0.213–4.313)	0.957
Laterality (left vs. right)	1.163 (0.502–2.696)	0.725	0.738 (0.161–3.390)	0.696
Operation (radical vs. partical)	0.371 (0.085–1.615)	0.186	0.407 (0.071–2.346)	0.315
Tumor size	1.061 (0.943–1.194)	0.324	13.307 (0.482–367.727)	0.126
T stage (T1–T2 vs. T3–T4)	3.832 (1.500–9.788)	0.005	8.089 (0.664–98.568)	0.126
N stage (N0 vs. N1)	5.556 (1.990–15.508)	0.001	1.340 (1.007–1.784)	0.045
M stage (M0 vs. M1)	13.719 (3.460–54.390)	<0.001	28.727 (3.079–267.990)	0.003
ISUP grade (1–2 vs. 3–4)	0.601 (0.195–1.851)	0.375	0.116 (0.006–2.175)	0.150
4EBP1 expression (low vs. high)	1.779 (0.765–4.133)	0.181	0.323 (0.072–1.453)	0.141
p-4EBP1 expression (low vs. high)	5.291 (1.477–18.959)	0.011	33.750 (1.883–604.899)	0.017
p-mTOR expression (low vs. high)	3.037 (1.253–7.362)	0.014	8.766 (1.212–63.424)	0.032
p-S6K expression (low vs. high)	1.791 (0.759–4.229)	0.183	0.752 (0.115–4.907)	0.766
p-MAPK expression (low vs. high)	1.393 (0.597–3.247)	0.443	1.504 (0.289–7.817)	0.628

PFS, Progression-free survival; Xp11.2 RCC, Xp11.2 translocation renal cell carcinoma; ISUP, International Society of Urological Pathology; 4EBP1, eukaryotic initiation factor 4E (eIF4E) binding protein 1; p-4EBP1, phosphorylated eukaryotic initiation factor 4E (eIF4E) binding protein 1; p-mTOR, phosphorylated mammalian target of rapamycin; p-S6K, phosphorylated ribosomal protein S6 kinase; p-MAPK, phosphorylated mitogen-activated protein kinase.

**Table 4:** Univariate and multivariate Cox regression analyses of OS in 36 enrolled adult Xp11.2 RCC patients.

Covariates	Univariate analysis		Multivariate analysis	
	HR (95%CI)	P value	HR (95%CI)	P value
Age at surgery	0.970 (0.920–1.023)	0.268	0.883 (0.756–1.032)	0.118
Sex (male vs. female)	0.569 (0.173–1.873)	0.353	2.676 (0.135–52.925)	0.518
Clinical manifestation (incidental vs. symptomatic)	0.726 (0.216–2.445)	0.606	0.057 (0.001–6044.600)	0.628
Laterality (left vs. right)	0.765 (0.223–2.621)	0.670	0.776 (0.038–15.792)	0.869
Operation (radical vs. partical)	0.034 (0.001–22.757)	0.309	36.308 (0.060–21828.088)	0.271
Tumor size	1.014 ()0.871–1.182	0.855	0.781 (0.536–1.138)	0.198
T stage (T1–T2 vs. T3–T4)	7.267 (1.737–30.397)	0.007	10.423 (0.312–348.158)	0.190
N stage (N0 vs. N1)	10.621 (2.264–49.813)	0.003	2.995 (0.320–27.975)	0.336
M stage (M0 vs. M1)	17.158 (3.349–87.910)	<0.001	19.849 (1.121–351.393)	0.042
ISUP grade (1–2 vs. 3–4)	0.209 (0.051–0.858)	0.030	4.637 (0.381–56.453)	0.229
4EBP1 expression (low vs. high)	1.476 (0.386–5.647)	0.570	1.177 (0.087–15.950)	0.902
p-4EBP1 expression (low vs. high)	6.735 (1.374–33.014)	0.019	56.111 (1.600–1967.704)	0.026
p-mTOR expression (low vs. high)	1.991 (0.597–6.638)	0.263	9.548 (0.773–117.949)	0.079
p-S6K expression (low vs. high)	1.391 (0.420–4.610)	0.589	0.346 (0.024–4.971)	0.435
p-MAPK expression (low vs. high)	1.189 (0.362–3.910)	0.775	1.188 (0.148–9.542)	0.871

OS, overall survival; Xp11.2 translocation renal cell carcinoma; ISUP, International Society of Urological Pathology; 4EBP1, eukaryotic initiation factor 4E (eIF4E) binding protein 1; p-4EBP1, phosphorylated eukaryotic initiation factor 4E (eIF4E) binding protein 1; p-mTOR, phosphorylated mammalian target of rapamycin; p-S6K, phosphorylated ribosomal protein S6 kinase; p-MAPK, phosphorylated mitogen-activated protein kinase.
