# Supplementary material for: Phosphorylated 4EBP1 is associated with tumor progression and poor prognosis in Xp11.2 translocation renal cell carcinoma
Source: Sci Rep. 2016 Mar 30;6:23594. doi: 10.1038/srep23594 (PMC4812307; doi:10.1038/srep23594)
Supplement: Supplementary Information [file srep23594-s1.pdf]

Phosphorylated 4EBP1 is associated with tumor progression and poor prognosis in Xp11.2 translocation renal cell carcinoma

Running title: p-4EBP1 is associated with tumor progression and poor prognosis in Xp11.2 RCC

Yuanyuan Qu[¶1,4], Rui Zhao[¶2], Hongkai Wang[1,4], Kun Chang[1,4], Xiaoqun Yang[3,4], Xiaoyan Zhou[3,4], Bo Dai[1,4], Yao Zhu[1,4], Guohai Shi[1,4] , Hailiang Zhang*[1,4], Dingwei Ye*[1,4]

[¶]Co-first authors

Supplementary Figures

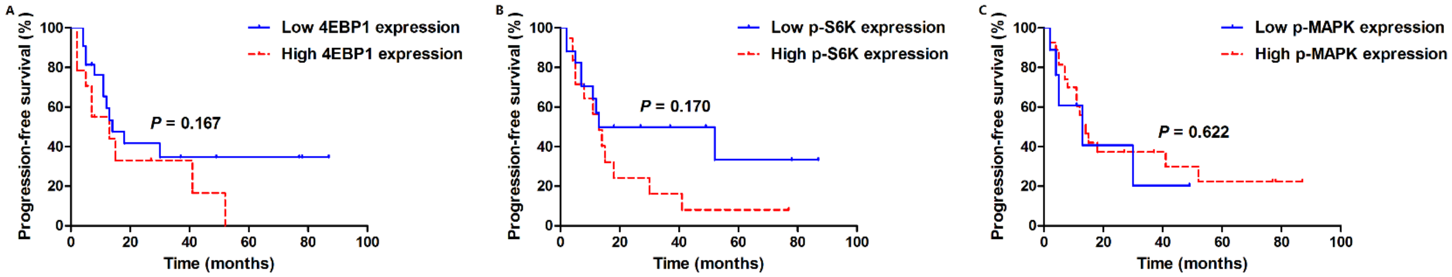

Supplemental Figure 1. Kaplan–Meier analysis of PFS curves in adult Xp11.2 RCC patients. (A) Stratified according to 4EBP1 expression (log-rank P = 0.167); (B) Stratified according to p-S6K expression (log-rank P = 0.170); (C) Stratified according to p-MAPK expression (log-rank P = 0.622). PFS, progression-free survival; Xp11.2 RCC, Xp11.2 translocation renal cell carcinoma; 4EBP1, eukaryotic initiation factor 4E (eIF4E) binding protein 1; p-S6K, phosphorylated ribosomal protein S6 kinase; p-MAPK, phosphorylated mitogen-activated protein kinase.

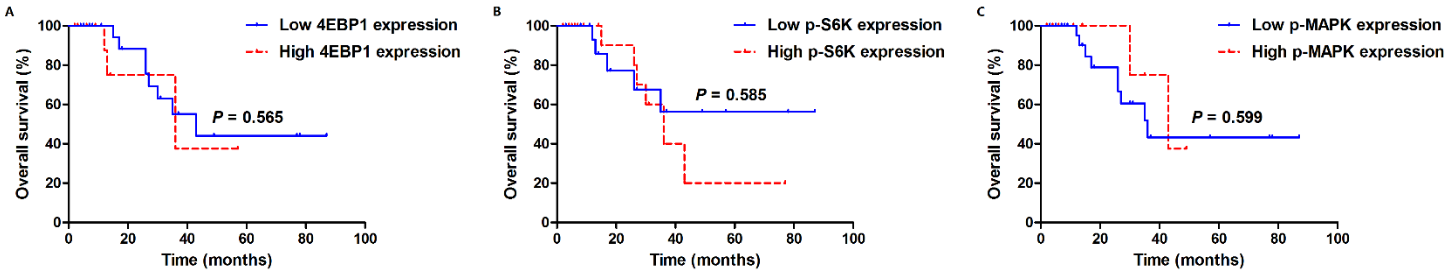

Supplemental Figure 2. Kaplan–Meier analysis of OS curves in adult Xp11.2 RCC patients. (A) Stratified according to 4EBP1 expression (log-rank P = 0.565); (B) Stratified according to p-S6K expression (log-rank P= 0.585); (C) Stratified according to p-MAPK expression (log-rank P= 0.599). OS, overall survival; Xp11.2 RCC, Xp11.2 translocation renal cell carcinoma; 4EBP1, eukaryotic initiation factor 4E (eIF4E) binding protein 1; p-S6K, phosphorylated ribosomal protein S6 kinase; p-MAPK, phosphorylated mitogen-activated protein kinase.

Supplementary Tables

Supplementary Table 1. Clinicopathological characteristics in relation to 4EBP1/p-mTOR expression status

**Variable**	4EBP1 expression		*P* value	p-mTOR expression		*P* value
	Low expression (n=22)	High expression (n=14)		Low expression (n=20)	High expression (n=16)	
Median age at surgery (y, range)	27.0 (14.0-63.0)	30.0 (14.0-40.0)	0.476	30.0 (14.0-63.0)	27.0 (14.0-47.0)	0.344
Sex (n, %)			0.143			0.877
Male	10 (45.5)	3 (21.4)		7 (35.0)	6 (37.5)	
Female	12 (54.5)	11 (78.6)		13 (65.0)	10 (62.5)	
Clinical manifestation (n, %)			0.204			0.221
Incidental	11 (50.0)	4 (28.6)		8 (40.0)	8 (50.0)	
Symptomatic	11 (50.0)	10 (71.4)		12 (60.0)	8 (50.0)	
Laterality (n, %)			0.494			1.000
Left	10 (45.5)	8 (57.1)		10 (50.0)	8 (50.0)	
Right	12 (54.5)	6 (42.9)		10 (50.0)	8 (50.0)	
Operation (n, %)			0.760			0.230
Radical nephrectomy	18 (81.8)	12 (85.7)		18 (90.0)	12 (75.0)	
Partial nephrectomy	4 (18.2)	2 (14.3)		2 (10.0)	4 (25.0)	
Median tumor size (cm, range)	5.1 (2.3-18.0)	6.7 (2.0-15.0)	0.234	6.4 (2.0-18.0)	5.5 (2.6-11.2)	0.433
T stage at presentation (n, %)			0.809			0.343
T1-T2	15 (68.2)	9 (64.3)		12 (60.0)	12 (75.0)	
T3-T4	7 (31.8)	5 (35.7)		8 (40.0)	4 (25.0)	
N stage at presentation (n, %)			0.697			0.878
N0	14 (63.6)	8 (57.1)		12 (60.0)	10 62.5()	
N1	8 (36.4)	6 (42.9)		8 (40.0)	6 (37.5)	

M stage at presentation (n, %)			0.465			0.720
M0	18 (81.8)	10 (71.4)		16 (80.0)	12 (75.0)	
M1	4 (18.2)	4 (28.6)		4 (20.0)	4 (25.0)	
ISUP grade (n, %)			0.361			0.720
1-2	6 (27.3)	2 (14.3)		4 (20.0)	4 (25.0)	
3-4	16 (72.7)	12 (85.7)		16 (80.0)	12 (75.0)	
Adjuvant therapy (n, %)			0.359			0.382
None	9 (40.9)	4 (28.6)		9 (45.0)	4 (25.0)	
Immunotherapy	4 (18.2)	1 (7.1)		3 (15.0)	2 (12.5)	
Targeted therapy	9 (40.9)	9 (64.3)		8 (40.0)	10 (62.5)	

Continued Supplementary Table 1. Clinicopathological characteristics in relation to p-S6K/p-MAPK expression status

	p-S6K expression			p-MAPK expression		
**Variable**	Low expression (n=17)	High expression (n=19)	*P* value	Low expression (n=27)	High expression (n=9)	*P* value
Median age at surgery (y, range)	28.0 (14.0-63.0)	31.0 (14.0-48.0)	0.978	30.9 (14.0-63.0)	25.2 (14.0-48.0)	0.227
Sex (n, %)			0.429			0.161
Male	5 (29.4)	8 (42.1)		8 (29.6)	5 (55.6)	
Female	12 (70.6)	11 (57.9)		19 (71.4)	4 (44.4)	
Clinical manifestation (n, %)			0.463			0.079
Incidental	6 (35.3)	9 (47.4)		9 (33.3)	6 (66.7)	
Symptomatic	11 (64.7)	10 (52.6)		18 (66.7)	3 (33.3)	
Laterality (n, %)			0.095			0.054
Left	11 (64.7)	7 (36.8)		16 (59.3)	2 (22.2)	
Right	6 (35.3)	12 (63.2)		11 (40.7)	7 (77.8)	
Operation (n, %)			0.455			0.606
Radical nephrectomy	15 (88.2)	15 (78.9)		23 (85.2)	7 (77.8)	
Partial nephrectomy	2 (11.8)	4 (21.1)		4 (14.8)	2 (22.2)	
Median tumor size (cm, range)	6.4 (2.0-18.0)	5.5 (2.6-10.5)	0.218	7.0 (2.6-18.0)	5.3 (2.6-10.5)	0.235
T stage at presentation (n, %)			0.345			1.000
T1-T2	10 (58.8)	14 (73.7)		18 (66.7)	6 (66.7)	
T3-T4	7 (41.2)	5 (26.3)		9 (33.3)	3 (33.3)	
N stage at presentation (n, %)			0.790			0.693
N0	10 (58.8)	12 (63.2)		17 (63.0)	5 (55.6)	
N1	7 (41.2)	7 (36.8)		10 (37.0)	4 (44.4)	
M stage at presentation (n, %)			0.858			1.000
M0	13 (76.5)	15 (78.9)		21 (77.8)	7 (77.8)	

M1	4 (23.5)	4 (21.1)		6 (22.2)	2 (22.2)	
ISUP grade (n, %)			0.532			0.355
1-2	3 (17.6)	5 (26.3)		5 (18.5)	3 (33.3)	
3-4	14 (82.4)	14 (73.7)		22 (81.5)	6 (66.7)	
Adjuvant therapy (n, %)			0.920			0.138
None	6 (35.3)	7 (36.8)		10 (37.0)	3 (33.3)	
Immunotherapy	2 (11.8)	3 (15.8)		2 (7.4)	3 (33.3)	
Targeted therapy	9 (52.9)	9 (47.4)		15 (55.6)	3 (33.3)	

4EBP1, eukaryotic initiation factor 4E (eIF4E) binding protein 1; p-mTOR, phosphorylated mammalian target of rapamycin; ISUP, International Society of Urological Pathology; p-S6K, phosphorylated ribosomal protein S6 kinase; p-MAPK, phosphorylated mitogen-activated protein kinase.
